# A Flexible 360-Degree Thermal Sound Source Based on Laser Induced Graphene

**DOI:** 10.3390/nano6060112

**Published:** 2016-06-07

**Authors:** Lu-Qi Tao, Ying Liu, Zhen-Yi Ju, He Tian, Qian-Yi Xie, Yi Yang, Tian-Ling Ren

**Affiliations:** 1 Institute of Microelectronics, Tsinghua University, Beijing 10084, China; taoluqi@126.com (L.-Q.T.); liuying_ly10@126.com (Y.L.); juzhy3rd@163.com (Z.-Y.J.); cheney_xie@126.com (Q.-Y.X.); 2 Tsinghua National Laboratory for Information Science and Technology (TNList), Tsinghua University, Beijing 100084, China; 3 Ming Hsieh Department of Electrical Engineering, University of Southern California, Los Angeles, CA 90089, USA; tianhe8832@163.com

**Keywords:** 360-degree sound source, graphene oxide, laser induced graphene, direct laser writing

## Abstract

A flexible sound source is essential in a whole flexible system. It’s hard to integrate a conventional sound source based on a piezoelectric part into a whole flexible system. Moreover, the sound pressure from the back side of a sound source is usually weaker than that from the front side. With the help of direct laser writing (DLW) technology, the fabrication of a flexible 360-degree thermal sound source becomes possible. A 650-nm low-power laser was used to reduce the graphene oxide (GO). The stripped laser induced graphene thermal sound source was then attached to the surface of a cylindrical bottle so that it could emit sound in a 360-degree direction. The sound pressure level and directivity of the sound source were tested, and the results were in good agreement with the theoretical results. Because of its 360-degree sound field, high flexibility, high efficiency, low cost, and good reliability, the 360-degree thermal acoustic sound source will be widely applied in consumer electronics, multi-media systems, and ultrasonic detection and imaging.

## 1. Introduction

With the development of nanomaterials and nanotechnologies, more and more flexible and novel electronic devices have been invented, such as flexible pressure and strain sensors [1,2,3,4], displays [5,6,7], touch screens [8], and energy storage devices [9,10,11]. In order to achieve an entirely flexible system, it is crucially important to develop a flexible sound source. The most common mechanism of conventional sound sources is via a piezoelectric part, by which an electrical signal is converted to a mechanical vibration. The piezoelectric part has no flexibility resulting in the limitation in a totally flexible electronic system. Compared to a conventional sound source, a thermal sound source can emit sound without any mechanical moving parts. When the alternating current is applied on the thermal sound source, it will heat up the air around the surface of the sound source, resulting in a pressure oscillation which is called the thermoacoustic effect [12]. Therefore, the thermal sound source can be applied in an entirely flexible system.

Some flexible thermal sound sources have been reported in recent years [13,14,15,16,17,18,19]. A multi-layer graphene sound source was first invented in 2011 [20] and demonstrates the potential of flexible sound sources. In 2012, some single-layer graphene sound sources were developed [21,22]. However, the fabrication of these graphene-based materials is based on chemical vapor deposition (CVD), which requires a lot of time for graphene growth and transferring. Recently, the direct laser writing (DLW) process has been proved to have great potential for fabricating graphene-based materials [23]. Some graphene-based devices have been developed based on laser induced graphene, such as transistors [24], resistive random access memory (RRAM) [25], supercapacitors [26,27,28], photodetectors [29], humidity sensors [30], and so on. However, a flexible 360-degree thermal sound source and related experiment has never been reported. 

In this study, we developed a time-efficient and large-scale 360-degree thermal sound source based on laser induced graphene. The foam-like structure of laser induced graphene is beneficial for emitting sound, because the pored and foam-like structure prevents heat leakage. The flexible sound source was attached to a cylinder so that it would generate a 360-degree sound wave. A theoretical model was built, and the experiment results fit well with the theoretical results. The results show that a 360-degree thermal sound source was successfully developed that it will be helpful for the realization of an entirely flexible system.

## 2. Experimental Procedure

### 2.1. Materials Preparation

Graphene oxide (GO) dispersion with a 2 mg/mL concentration was provided by XFNANO Materials Tech Co., Ltd. (Nanjing, China). A 10 mL GO solution was coated on the polyethylene terephthalate (PET) substrate. The GO solution was placed under a bake lamp at 50 °C for 4 h, and an approximately 1-μm-thick GO sheet was formed.

### 2.2. Fabrication of the 360-Degree Thermal Sound Source

**Figure 1:**
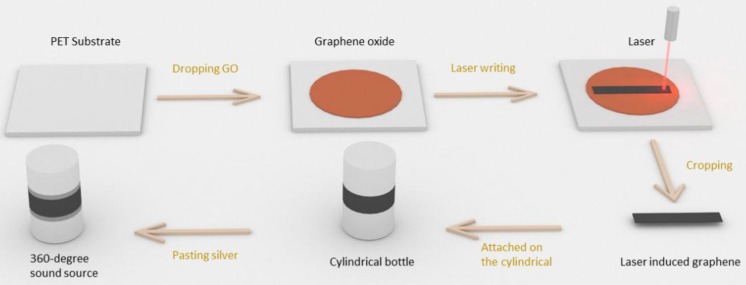
Fabrication process of the 360-degree thermal sound source.

The fabrication process of the 360-degree thermal sound source is shown in Figure 1. The GO solution was coated on the PET substrate, and a GO sheet was formed on the PET substrate. Then, GO/PET was irradiated by a potable continuous laser platform with a wavelength of 650 nm and power of 0.13 mW. The focused spot of the laser was approximately 100 μm, and the power density of the laser was 16.55 mW/mm^2^. The scanning speed was only 7 mm/s so that the overlap would overcome the Gaussian light distribution in each line. The laser was controlled by two stepper motors so that it could move in the X-Y plane freely. Custom-designed pictures could be imported into the software, and patterned graphene was formed by the irradiation of the laser. After the fabrication, the thermal sound source was cut into a special shape and attached to the surface of a cylinders with diameters of 25 mm, 15 mm, and 4 mm. The device was wired out by copper wire using silver paste.

### 2.3. Performance Testing of the 360-Degree Thermal Sound Source

**Figure 2:**
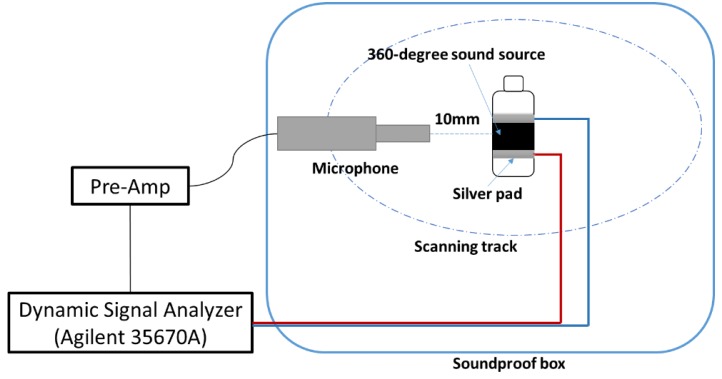
Schematic illustration of the testing platform of 360-degree thermal sound source.

Figure 2 shows the experimental platforms to test the performance of the 360-degree thermal sound source. The acoustic signal was received by a 10-mm-long high-sensitive microphone (Earthworks M50, Earthworks Audio, Milford, NH, USA), which was connected with a dynamic signal analyzer (Agilent 35670A, Agilent Technologies Inc., Palo Alto, CA, USA). The dynamic signal analyzer was used to produce a swept signal to drive the 360-degree thermal sound source and record the value of the sound pressure level. The acoustic testing was performed in a soundproof box (1.0 × 1.0 × 0.5 m^3^) full of sound absorbing sponges to avoid echo and noise from outside.

## 3. Results and Discussions

**Figure 3:**
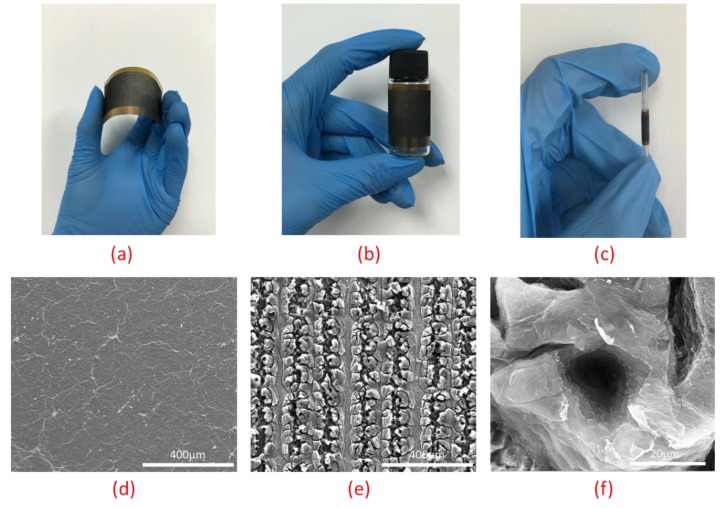
The morphology and structure of the laser induced graphene thermal sound source. (**a**) The flexible thermal sound source in hand. (**b**) The 360-degree thermal sound source attached to a cylindrical bottle. (**c**) The 360-degree thermal sound source attached to an ultrathin stick. (**d**) The surface profile of graphene oxide (GO) under scanning electron microscope (SEM). (**e**) The SEM image of laser induced graphene under low magnification. (**f**) The SEM image of laser induced graphene under high magnification.

Figure 3a shows the photos of the 360-degree thermal sound source. The device had excellent flexibility. As shown in Figure 3b,c, the device could be attached to the surface of a cylindrical bottle and an ultrathin stick by the double-side tape. The strain would not break the laser induced graphene because the strain was not in the direction of the graphene stripes. It was hard to decrease the diameter further, because the adhesive power was not enough to support the elastic force. The morphology and structure of GO sheets before and after the laser’s irradiation were observed with scanning electron microscope (SEM). The original structure of GO without irradiating was flat and uniform with little roughness, as shown in Figure 3d. The resistance of the original GO is 580 MΩ. The morphology of laser induced graphene under low magnification is illustrated in Figure 3e, where we can clearly see the scanning track of the laser. The laser induced-graphene stripes are parallel to the central axis of the bottle and the stick. Pored and foam-like structures could be identified under high magnification, as shown in Figure 3f, and this is mainly attributed to the local high temperature resulting in the film’s expansion by gasification of oxygen species. Three samples with diameters of 25 mm, 15 mm, and 4 mm were fabricated, and the resistances of them were 312 Ω, 518 Ω and 1.8 kΩ.

**Figure 4:**
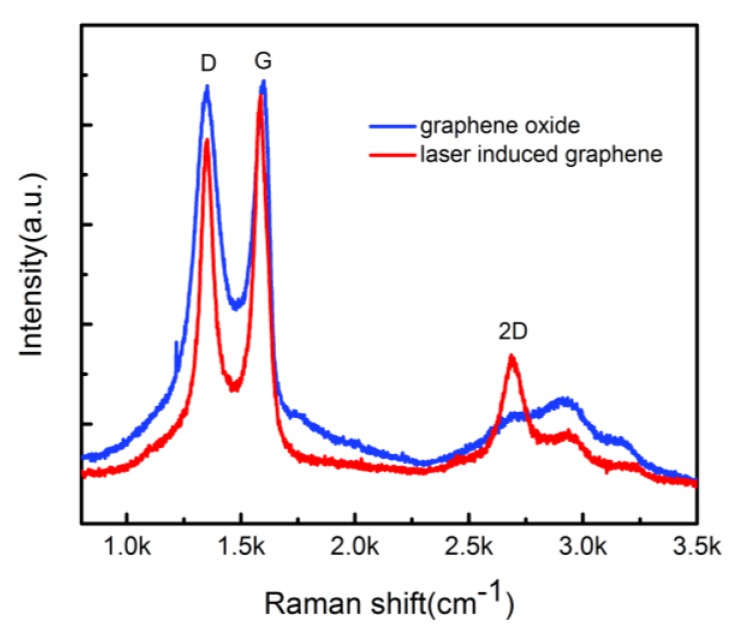
The Raman spectrum of the graphene oxide (blue line) and laser induced graphene (red line).

Raman spectrum was performed by choosing a single laser induced graphene spot to further research the characteristics of laser induced graphene. As shown in Figure 4, GO had the typical D, G, and amorphous 2D bands. It was noticed that the D band had a clear decline after laser direct writing, because C-oxygen functional groups were broken and the C–C bond was recombined. Moreover, the 2D band was clearly formed, meaning that multi-layer graphene was generated.

**Figure 5:**
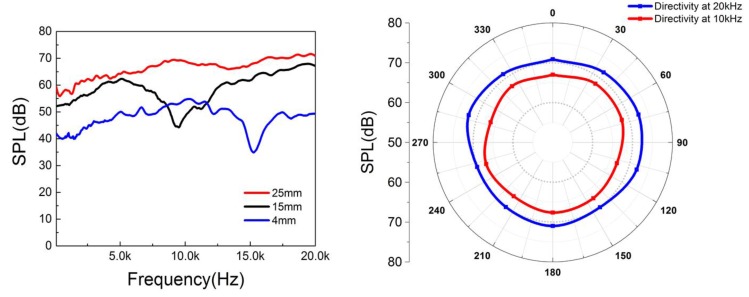
Performance testing of the 360-degree thermal sound source. (**a**) The output sound pressure level *vs.* the frequency. (**b**) The directivity of the thermal sound source.

The performances of the 360-degree thermal sound sources with diameters of 25 mm, 15 mm, and 4 mm were measured and are shown in Figure 5a. The sound frequency ranges from 100 Hz to 20 kHz. The sound pressure was recorded at a distance of 10 mm, and the curve was normalized with an input power of 1 W/cm^2^. It was noticed that the thermal sound source had a better performance in the high-frequency range compared with that in the low-frequency range. This kind of phenomenon is in agreement with other thermal sound sources [31]. We can notice that a sharp dip appears at a certain frequency when the diameter is smaller, and a gentle dip appears when the diameter is larger. This phenomenon may be a result of wave interference. Because our device was not a point source, interference would appear at some certain frequencies, which is related to the diameter of our device. From Figure 5b, the directivity of the sound source with a diameter of 25 mm was tested at frequencies of 10 kHz and 20 kHz. We found that the directivity was approximately omnidirectional, and it was quite different from planar sound sources [20].

A theoretical model was built to explain this phenomenon. The panorama of thermal acoustic was built by Arnold *et al.* [12]. When laser induced graphene is heated by an alternating current, the temperature of the surrounding air varies periodically, causing the air to expand and contract periodically. This process occurs within a range of several wavelengths. Then, the variation in temperature no longer exists, and the periodic movement of air changes into a sound wave. The heat transfer into air is calculated using equations in [13]:

(1) $${\dot{Q}}_{fluid}=\frac{\left(2+2\sqrt{2}\xi +2\xi^{2}+\sqrt{2}\xi^{3}\right)}{4{\left(1+\sqrt{2}\xi +\xi^{2}\right)}^{2}}\cdot \frac{P_{e}}{1+\chi }$$

where $\xi =\sqrt{\frac{\pi }{2}}\cdot \frac{\rho_{s}C_{s}d_{s}}{\sqrt{\rho_{air}C_{v,air}k_{air}}+\sqrt{\rho_{PI}C_{v,PI}k_{PI}}}\cdot f^{1/2}$*,*$\chi =\sqrt{\frac{\rho_{PI}C_{v,PI}k_{PI}}{\rho_{air}C_{v,air}k_{air}}}$*.* ρ*,* $C_{v}$*,* k is the density, capacity and heat transfer coefficient of these materials. Additionally, the relation between sound pressure and heat is given in [13]:

(2) $$p_{rms}=\frac{m_{air}\cdot f}{2\sqrt{2}C_{p}T_{0}r}\cdot {\dot{Q}}_{fluid}$$

**Figure 6:**
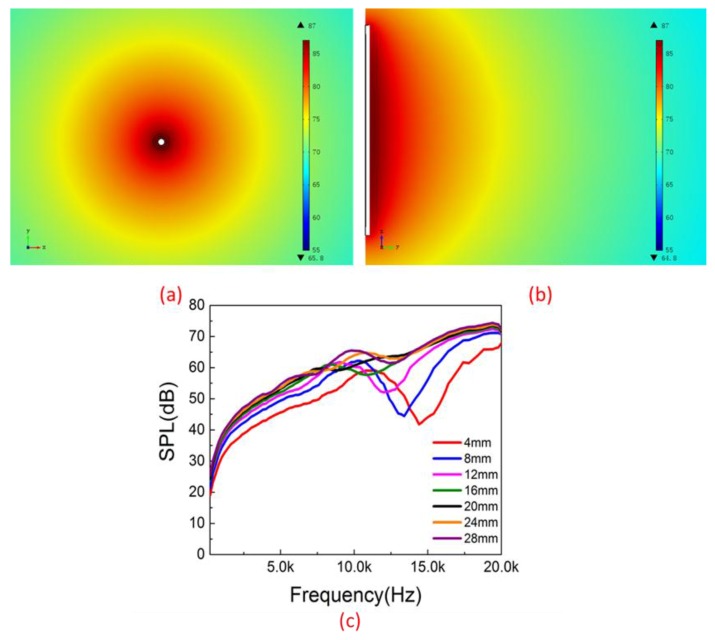
Simulation of the sound field for the device. (**a**) The sound field of horizontal plane of the device working at 20 kHz, showing great uniformity at 360 degrees. (**b**) The sound field of the vertical plane of the device working at 20 kHz. (**c**) The theoretical sound pressure level (SPL) *vs*. frequencies ranging from 100 Hz to 20 kHz.

Based on these points, a simulation model was established using multi-physical field and finite element analysis software. The device in the model is similar to the experimental device but is not exactly the same, because the pored and foam-like structures could not be reflected in the simulation model, so the simulation results may be slightly different from the experimental results. Figure 6 demonstrates the simulation results of this device. The sound field of the horizontal plane of the device working at 20 kHz shows great uniformity (Figure 6a). The sound field of the vertical plane of the device is also shown (Figure 6b), and it indicates that the sound pressure would decrease when the distance increased. The simulation results also provide the relations between the sound pressure level (SPL) and the frequency (Figure 6c). The dips are sharper and shift towards low frequencies when the diameters are smaller than 20 mm, and the dips become subdued when the diameters are larger than 20 mm. The positions of the dips are similar to the experimental results and prove the existence of wave interference.

## 4. Conclusions

In summary, we demonstrated a flexible 360-degree thermal sound source based on laser induced graphene. The focused 650-nm laser converted GO into foam-like laser induced graphene. The foam-like structure is beneficial for emitting sound by preventing heat leakage in the structure. Moreover, the patterning and reduction processes can be realized at the same time. The sound pressure level has been shown from 100 Hz to 20 kHz. A theoretical model is established to analyze the sound field, and the theoretical results are in good agreement with the experimental results. Due to its 360-degree sound field, high flexibility, high efficiency, low cost, and good reliability, the 360-degree thermal acoustic sound source will be widely applied in consumer electronics, multi-media systems, and ultrasonic detection and imaging.
